# Kikuchi-Fujimoto Disease With Systemic Lupus Erythematosus and Systemic Sclerosis Overlap: A Unique Clinical Presentation

**DOI:** 10.7759/cureus.44986

**Published:** 2023-09-10

**Authors:** Muhammad Burhan Majeed Rana, Minahil Fatima, Iqra M Rana, Muhammad Haseeb ul Rasool, Hazem Abosheaishaa, Adriana Abrudescu, Sabiha Bandagi

**Affiliations:** 1 Internal Medicine, Queens Hospital Center, New York City, USA; 2 Internal Medicine, Services Hospital Lahore, Lahore, PAK; 3 Internal Medicine, Allama Iqbal Medical College, Lahore, PAK; 4 Medicine, Icahn School of Medicine at Mount Sinai, Queens Hospital Center, New York City, USA; 5 Internal Medicine, Icahn School of Medicine at Mount Sinai, Queens Hospital Center, New York City, USA; 6 Internal Medicine/Gastroenterology, Cairo University, Cairo, EGY; 7 Rheumatology, Icahn School of Medicine at Mount Sinai, Queens Hospital Center, New York City, USA

**Keywords:** systemic sclerosis, systemic lupus erythematosis, kikuchi-fujimoto disease and systemic lupus erythematosus, kikuchi-fujimoto disease, membranous nephropathy

## Abstract

Kikuchi-Fujimoto Disease (KFD), or histiocytic necrotizing lymphadenitis (HNL), is a rare self-limiting disorder presenting with fever and swollen lymph nodes. It is characterized by the focal proliferation of reticular cells, the presence of nuclear debris, and histiocytes. In advanced cases, it can present with hepato-splenomegaly and generalized lymphadenopathy. Historically, it has been associated with viral infections, as it frequently was found to be associated with upper respiratory symptoms. Alternative explanations include the immune response of T-cells leading to alteration in CD8-positive T-cell-mediated cell apoptosis. It is also speculated that KFD can be associated with rheumatological autoimmune diseases. We present a case of a 21-year-old African American female with a known diagnosis of systemic lupus erythematosus (SLE)-systemic sclerosis (SS) overlap presented with febrile lymphadenopathy and was diagnosed to have HNL on histological exam of lymph node biopsy.

## Introduction

Kikuchi-Fujimoto disease (KFD), or histiocytic necrotizing lymphadenitis (HNL), is a rare, benign, self-limiting idiopathic disorder frequently presenting with the symptoms of fever and swollen lymph nodes. Though previously prevalent in young Asian women, KFD is now increasingly being diagnosed globally in all age groups and ethnicities. We discuss a 21-year-old African American female with a known diagnosis of systemic lupus erythematosus (SLE)-systemic sclerosis (SS) overlap who presented with febrile lymphadenopathy, the work-up leading to diagnosis, as well as a literature review on this particularly unique presentation which, to the best of our knowledge, is the first reported case of KFD in the setting of known KFD-SLE overlap.

## Case presentation

**Figure 1:**
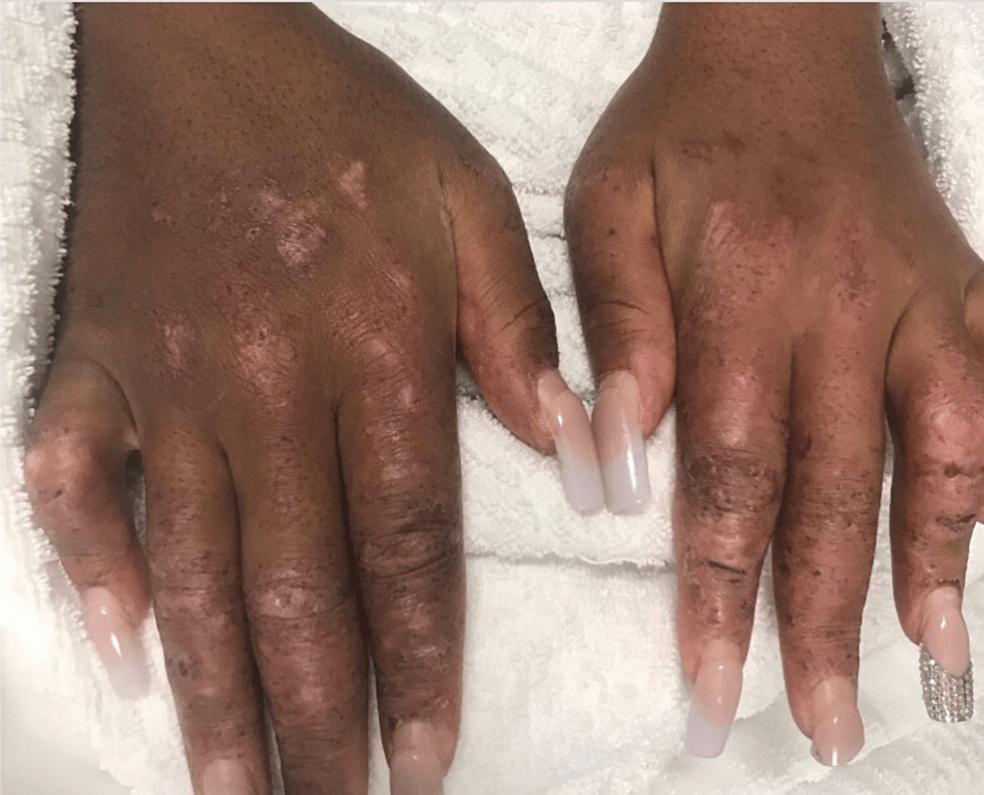
Physical exam of hands

**Figure 2:**
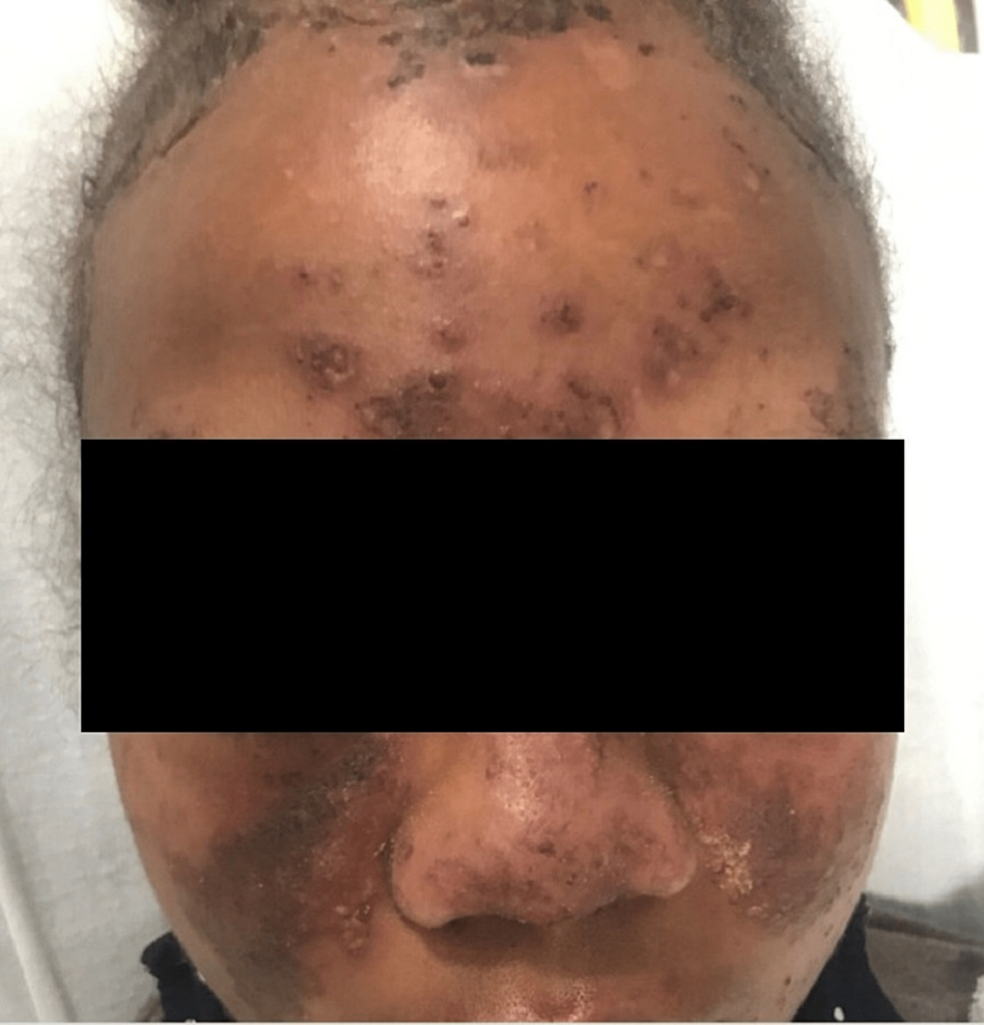
Physical exam revealing facial rash

A 21-year-old African American female with a past medical history of systemic lupus erythematosus, scleroderma, and glucose-6-phosphate dehydrogenase (G6PD) deficiency, presented initially with worsening and diffuse skin rash (including malar pattern), sclerodactyly, Raynaud’s phenomenon, high fever, pancytopenia with evidence of hemolysis, hematuria, and proteinuria after a trip to Costa Rica 2 weeks ago. Her presenting vitals include a blood pressure of 96/69, heart rate of 122 beats per minute (BPM), febrile to 102.2 F, and saturating to 97% on room air. Her physical exam findings are demonstrated in Figure 1 and Figure 2.

Lab results (Table 1) demonstrated elevated erythrocyte sedimentation rate (ESR), C-reactive protein (CRP), low C3 complement level, high anti-DNA antibiotics, elevated scleroderma (SCL) antibodies, elevated Sjogren’s SS A Ab (RO) and proteinuria. Further workup confirmed low G6PD levels and hydroxychloroquine could not be started. The patient was started on prednisone and azathioprine for SLE and SS and was suggested to continue to follow up in an outpatient clinic. However, the patient presented again to the emergency department (ED) with complaints of fevers, joint pains, shortness of breath, as well as bilateral tender parotid gland enlargement, and cervical lymphadenopathy for 5 days. Her vitals in the ED included a heart rate of 135 BPM, blood pressure of 80/54, febrile to 101.8 F, and saturating to 97% on room air.

**Table 1:** Table Illustrating lab results x implies a test not done on a particular admission The admission number mentioned presents the sequential admission to inpatients on three separate occasions

Lab values (reference range)	Admission # 1	Admission # 2	Admission # 3
White cell count (4.8 - 10.8 x 10(3)/mcL)	4.01	1.61	13.35
Erythrocyte sedimentation rate (ESR) (0-20 mm/hr)	83	22	132
High-reactive C-reactive protein (<=5mg/L)	9.30	1.20	130.60
Blood cultures	Negative	Negative	Negative
Urine cultures	Negative	Negative	Negative
C3 complement (81 - 157 mg/dL)	67	70	117
C4 complement (13 - 39 mg/dL)	15	20	18
Anti-DNA antibody, double-stranded (<=29 IU/mL)	46	22	15
Anti-Smith antibody (<=0.9 AI)	7.2	1.3	0.4
Scleroderma antibodies/SCL 70 Ab (<=0.9 AI)	>8.0	>8.0 (chronic value)	x
Sjogren’s SS A Ab (RO) (<=0.9 AI)	>8.0	3.6	x
Sjogren’s SS B Ab (LA) (<=0.9 AI)	<0.2	<0.2	x
Protein on urinalysis (negative mg/dL)	30	300	100
24-hour quantitative urine protein (<150 mg/24 hrs)	x	2460	x

The initial computerized tomography (CT) of neck soft tissue with contrast revealed diffuse enlargement of bilateral parotid glands, as well as non-specific centimeter/sub-centimeter lymph nodes, seen diffusely throughout the neck without significant enlargement or necrosis. The relevant lab results for this second admission are mentioned in Table 1, reflecting improved ESR, CRP, and worsened proteinuria. The patient was admitted to the medical floor with the initial impression of sepsis of unknown origin and possible SLE flare.

Ear, nose, and throat (ENT) surgery and rheumatology were consulted. ENT recommended that the patient had bilateral parotitis and sialadenitis, and recommended conservative management. Rheumatology recommended starting on IV methylprednisolone at 0.5 mg/kg twice daily dosing while serology results were awaited. Significant labs during this admission included progressive worsening leukopenia despite IV steroid exposure with febrile neutropenia and severe proteinuria. Total white blood cell count and absolute neutrophil count were 1.61 and 1.18 x 10(3)/mcL respectively as well as 24-hour quantitative urine protein was 2460 mg/24 hours. Hematology was consulted for said findings with concern for hemophagocytic lymphocytosis; however, it was recommended that once the patient improved clinically, she should get a bone marrow biopsy in outpatient settings. Nephrology recommended performing kidney biopsy in the outpatient setting as well. After showing improvement, the patient was discharged on oral steroids and antibiotics.

**Figure 3:**
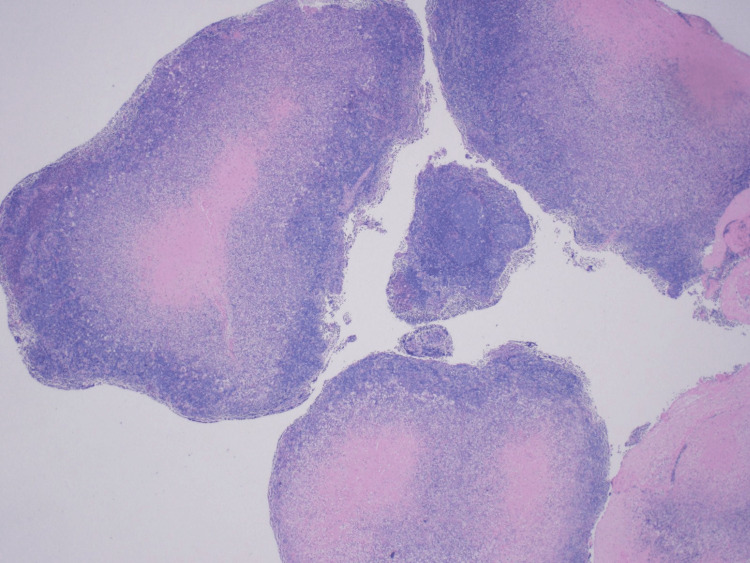
Histology of excisional lymph node biopsy revealing central necrosis with surrounding histiocytes, dendritic cells, and abundant karyorrhectic debris (magnification 20x)

**Figure 4:**
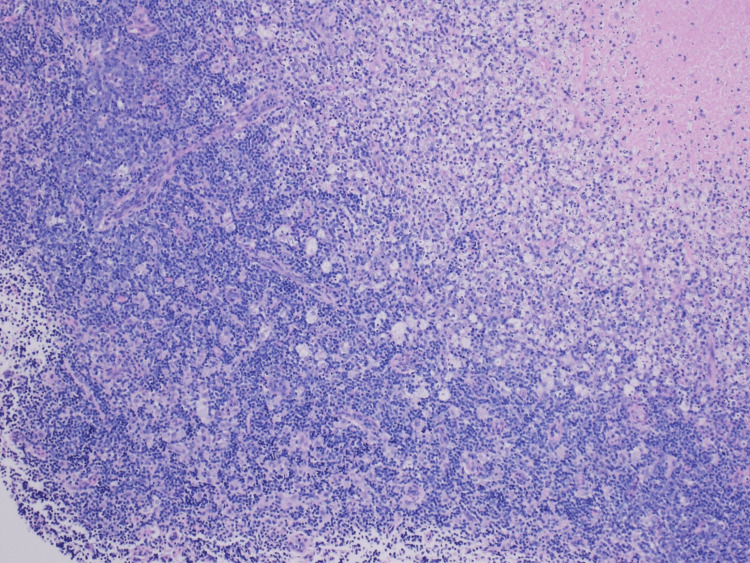
Histology of excisional lymph node biopsy revealing central necrosis with surrounding histiocytes, dendritic cells, and abundant karyorrhectic debris (magnification 100x)

**Figure 5:**
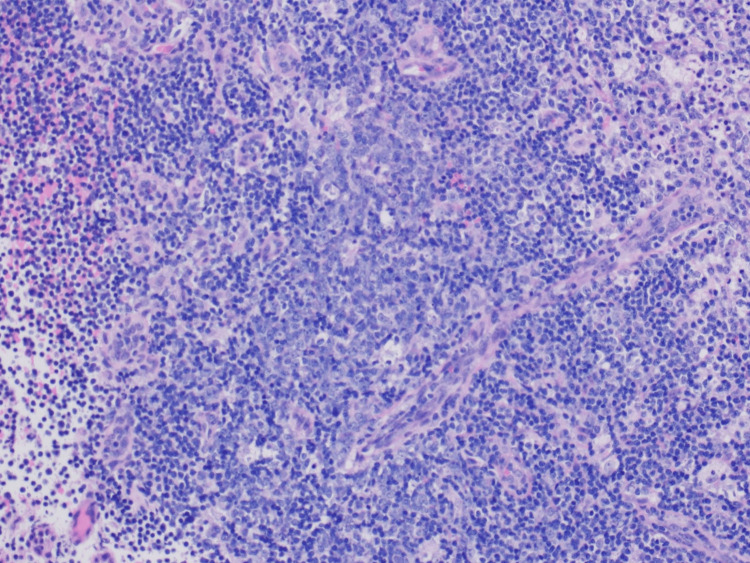
Histology of excisional lymph node biopsy revealing central necrosis with surrounding histiocytes, dendritic cells, and abundant karyorrhectic debris (magnification 200x)

However, the patient presented again to the ED with complaints of fever, back pain, and neck pain 2 weeks later. She was noted to have a soft, immobile supraclavicular mass of approximately 4 cm in diameter with warmth, tenderness, and erythema. She was again found to be febrile to 101 F and tachycardic to 116 BPM. CT of neck soft tissue showed interval development of central necrosis within multiple enlarged right cervical lymph nodes as well as a large left supraclavicular lymph node collection measuring 4.6 cm x 4.2 cm, most consistent with necrotizing lymphadenitis. It was suggested that Hodgkin’s lymphoma can have a similar appearance. The patient was re-admitted to the medicine service for close monitoring and further workup of new necrotizing lymphadenitis versus less likely lymphoma. ENT was consulted. Fine needle aspiration (FNA) was performed on the left supra-clavicular lymph node which resulted in many acute inflammatory cells admixed with lymphocytes and macrophages consistent with the acute inflammatory process. A follow-up excisional lymph node biopsy was performed next which revealed central necrosis with surrounding histiocytes, dendritic cells, and abundant karyorrhectic debris (Figures 3-5).

The final report read: fragments of lymphoid tissue showing central necrosis with predominantly macrophage infiltrates. Few preserved benign reactive lymphoid follicles were observed. CD30 reveals few reactive immunoblasts. BCL-2 is negative for those benign lymphoid follicles. CD15 highlights some neutrophils reactive to the above necrosis. The overall findings are suggestive of a benign reactive lymphoid tissue with necrosis, and the differential diagnosis includes Kikuchi disease, lupus lymphadenitis, infectious lymphadenitis, or other inflammatory processes. Clinical correlation is recommended. In addition, 10-color flow cytometry analysis for lymphoproliferative neoplasms was done which was negative for B-cell or T-cell lymphomas. The immunophenotypic analysis was significant for T-cells (64.5 % of the total) showing a CD4:CD8 ratio of 2.29:1. Based on the physical finding and the lab findings, she was diagnosed to have KFD, or HNL. She was started on mycophenolic acid and methylprednisolone, on which she was eventually discharged to follow up with rheumatology and nephrology clinics. She was found to have membranous glomerulopathy on renal biopsy and is currently following up with a nephrology and rheumatology clinic for continued management.

## Discussion

KFD is a benign and rare lymphadenitis of unknown pathogenesis described as the focal proliferation of reticular cells, the presence of nuclear debris, and histiocytes. It predominantly affects young women with a mean age of 20-30 but men and older women may also be affected [1]. It typically presents as swollen, tender lymphadenopathy in the neck with fever and night sweats with a minority also experiencing weight loss, chills, malaise, vomiting, sore throat, and gastrointestinal symptoms [2]. Generalized lymphadenopathy, splenomegaly, and hepatomegaly were observed in a small number of cases as well. Lab findings are usually non-specific and may show mild leukopenia, raised ESR, neutropenia, thrombocytopenia, anemia, elevated liver transaminases, or increased lactate dehydrogenase (LDH) [3,4]. Fever and lymphadenopathy are the most relevant manifestations [5].

To date, the etiology of the disease remains a mystery with multiple hypotheses explaining different possibilities. A viral pathology, such as an infection with Epstein-Barr virus (EBV), herpes simplex virus-1 (HSV-1), human papillomavirus (HPV), hepatitis B, or parvovirus has been speculated to be the most likely cause based on its association with an upper respiratory prodrome, atypical lymphocytosis, a lack of neutrophilic response, and a failure to improve with antibiotics [6,7]. However, no definite conclusion could be drawn. An alternative explanation suggests an immune response to T-cells. Histopathological studies have shown that the central mechanism of cell destruction appears to be apoptosis mediated by CD8-positive T-cells [8-10]. Morphological findings on Electron microscopy of the lymph nodes were also in line, showing the typical characteristic of an apoptotic cell including nuclear chromatin condensation and fragmentation along the nuclear membrane with intact organelles and histiocytes phagocytosing karyorrhectic debris.

Due to the possible immune nature of KFD, it is no surprise that the association of KFD with rheumatological autoimmune diseases has been well-documented in the literature, with the KFD diagnosis established before, during, and after SLE and SS [11-25]. Although the first case of KFD is traced to 1972 in Japan where it was described almost simultaneously by Kikuchi and Fujimoto, more and more cases are now being diagnosed in the Western world as well [26,27].

To the best of our knowledge, this is the first case of Kikuchi disease in a diagnosed patient of SLE-SS overlap syndrome. While evaluating our patient, the possible differential diagnosis included lupus lymphadenitis, malignant lymphoma, bacterial and viral infections, atypical mycobacterial tuberculosis, and KFD.

Due to the overlapping nature and clinical presentation of the above-mentioned differentials, fine needle biopsy followed by excisional biopsy was performed. Although the biopsy report came back negative for lymphoma, a feared outcome, the histopathology suggested Kikuchi or lupus lymphadenitis as the most likely diagnosis. The differentiation of lupus lymphadenitis and KFD is particularly challenging as they can have very similar clinical presentation and nodal histology. Because of this similarity, some authors have gone as far as to suggest that KFD may be a clinical feature of the temporary manifestation of lupus lymphadenitis. However, several isolated cases of KFD support the fact that KFD and SLE are independent entities [26-29].

The most common histological findings in KFD are a proliferation of histiocytes, foci of coagulative necrosis with abundant karyorrhexis, cellular debris, and apoptotic paracortical necrosis leading to a distorted nodal architecture [30,31]. The absence of neutrophils, eosinophils, hematoxylin bodies, and plasma cells further solidifies the diagnosis and favors KFD over lupus lymphadenitis [32]. A deeper analysis of the immunophenotype shows a predominance of T-cells in contrast to B-cells, with a prevalence of CD8 + T-cells.

In our patient, similar findings were seen. The lymphoid tissue showed central necrosis with predominantly macrophage infiltration. Neutrophils were scarce with the absence of DNA deposition in blood vessel walls, vasculitis around the necrotic foci as well as hematoxylin bodies (basophilic clusters pathognomonic for SLE) which led away from the possible diagnosis of lupus lymphadenitis. At the same time, a predominant population of T-cells (64.5% of total) > B-cells weighed into KFD as the probable diagnosis. However, it must be noted that histopathology alone may not be enough for a definitive diagnosis and clinical correlation must be made. One of the most important clinical features of KFD is its self-limiting nature. 64-80% of cases resolve within 1-4 months without any medical treatment with a small relapse rate of 1-4% [33]. Hence, long-term clinical follow-up is necessary.

## Conclusions

This case adds to the body of evidence suggesting the strong association of KFD with connective tissue disorders. Although no clear cause has yet been identified, the coexistence or overlap of KFD in these patients brings up the question of whether they have a common etiology or are part of the same spectrum. Despite similar clinical presentations, which can be a great challenge for clinicians, it further strengthens the importance of reaching an accurate diagnosis, as it can greatly impact patient care and wellness.
